# Supplementary material for: Grape Lipidomics: An Extensive Profiling thorough UHPLC-MS/MS Method
Source: Metabolites. 2021 Nov 30;11(12):827. doi: 10.3390/metabo11120827 (PMC8706896; doi:10.3390/metabo11120827)
Supplement: Supplementary file 1 [file metabolites-11-00827-s001.zip › Supplementary Material/Supplementary_Figures_20211117_R1.pptx]

## Slide 1
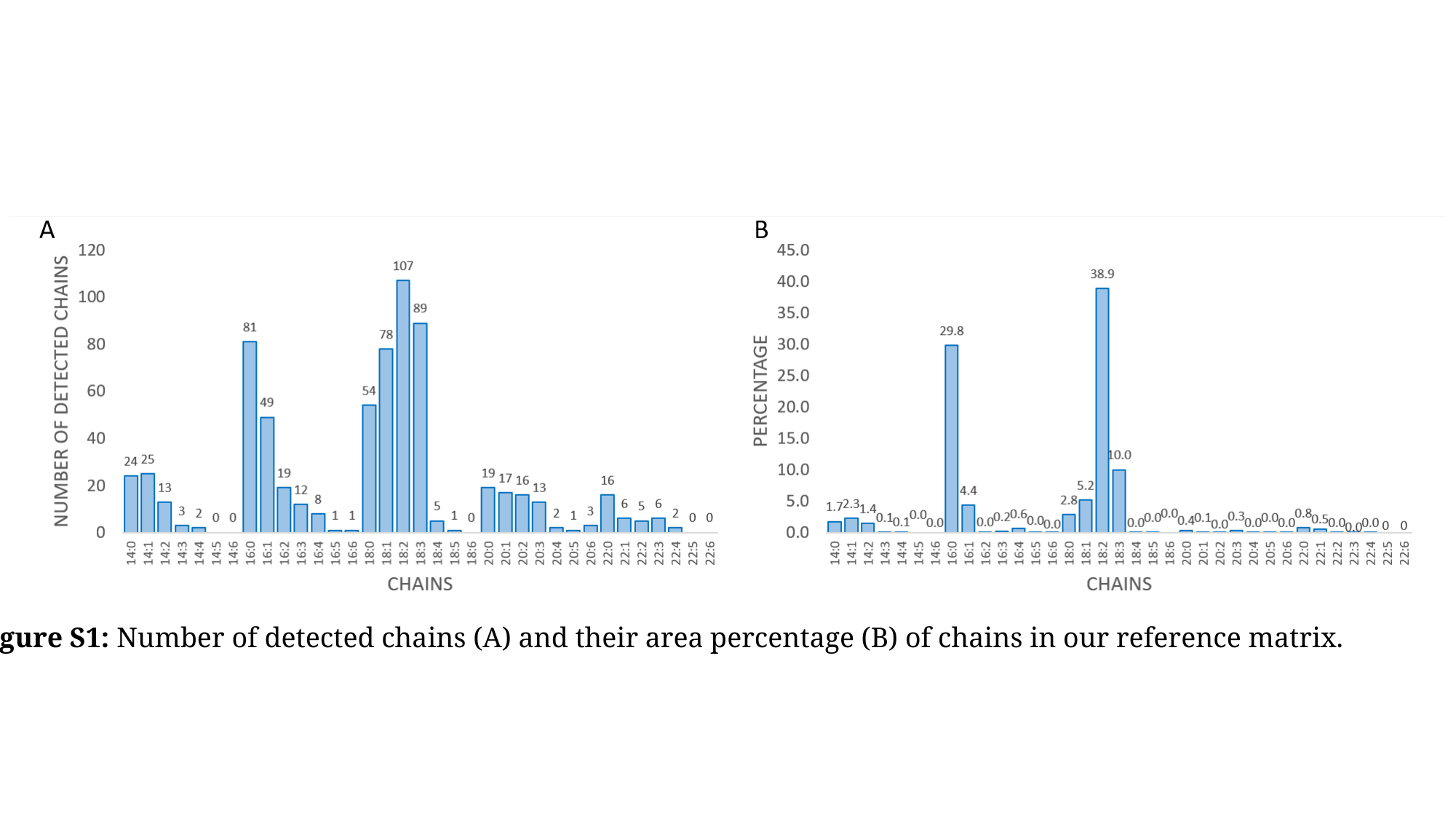

Figure S1: Number of detected chains (A) and their area percentage (B) of chains in our reference matrix.

## Slide 2
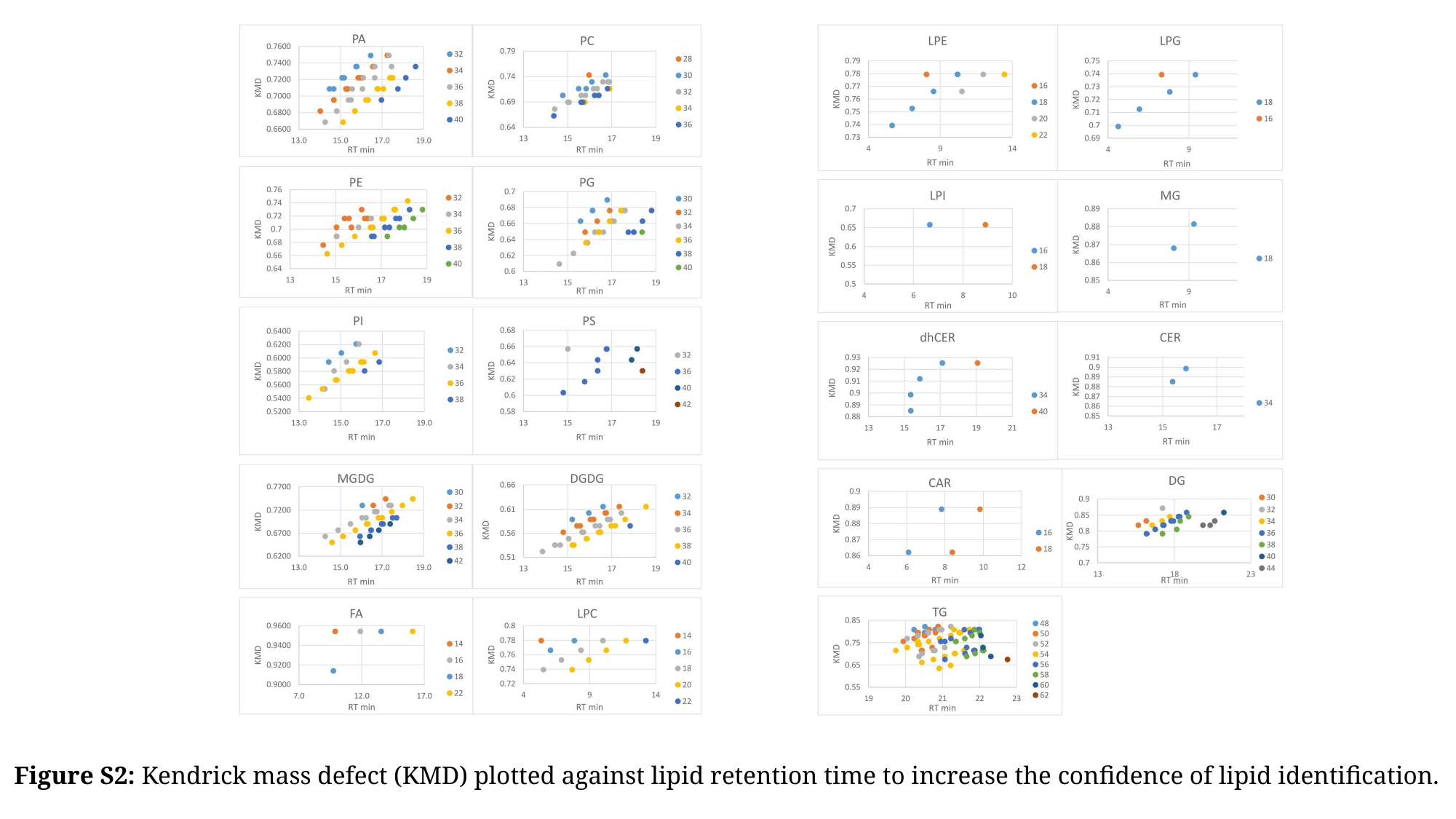

Figure S2: Kendrick mass defect (KMD) plotted against lipid retention time to increase the confidence of lipid identification.

## Slide 3
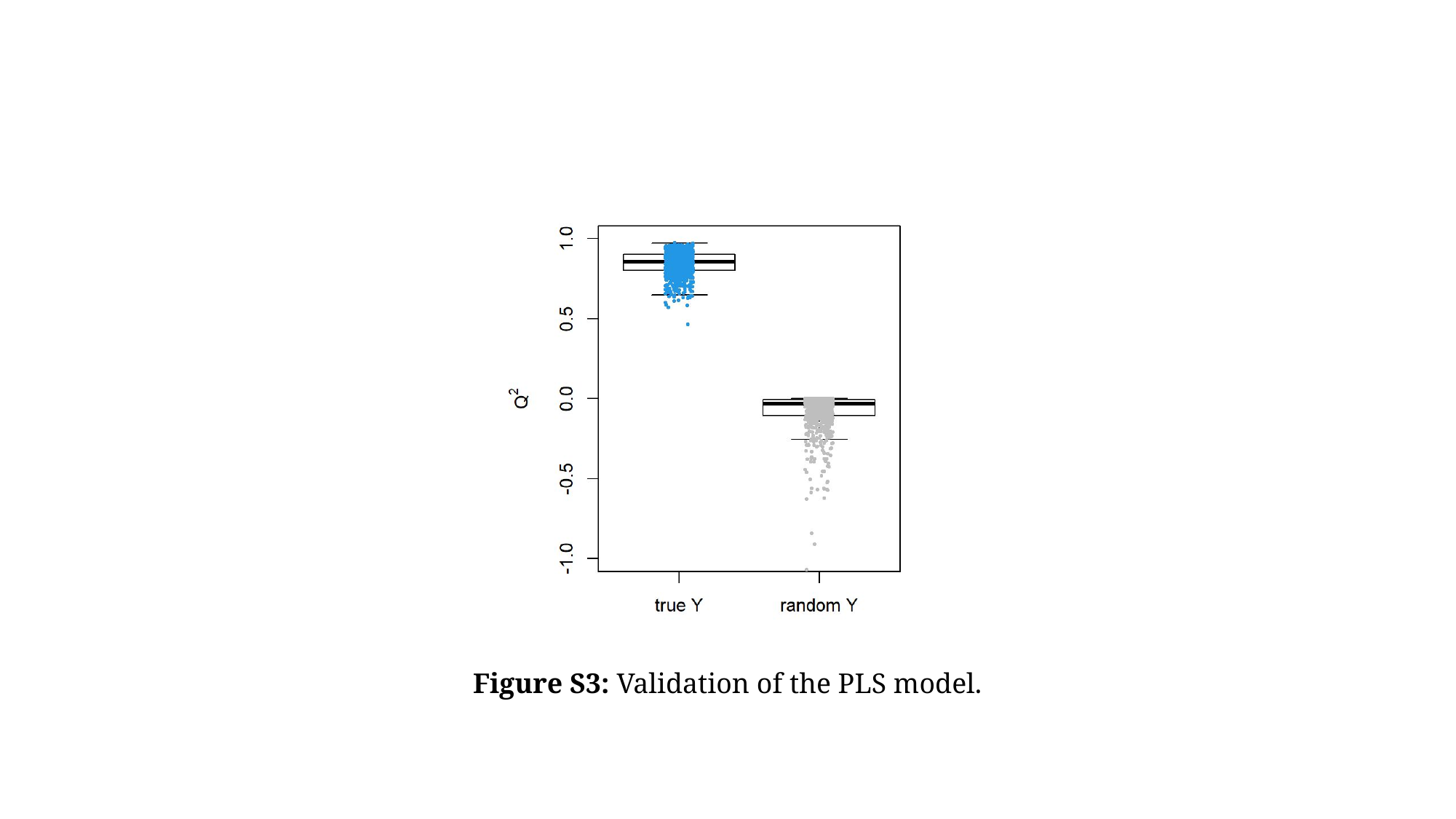

Figure S3: Validation of the PLS model.
